# Supportive and palliative care needs among older adults in India: an estimation using a nationally representative survey

**DOI:** 10.1186/s12904-024-01604-2

**Published:** 2024-11-30

**Authors:** Terrymize Immanuel, Naveen Salins, Benson Thomas M, Jenifer Jeba Sundararaj, Roop Gursahani

**Affiliations:** 1 SRMIST (Deemed to be University), School of Public Health, Kattankulathur, Chennai, Tamil Nadu 603203 India; 2 Department of Palliative Medicine and Supportive Care, Kasturba Medical College Manipal, Manipal Academy of Higher Education, Manipal, 576104 India; 3 Department of Palliative Medicine, Christian Medical College, Vellore, Tamil Nadu India; 4 Department of Neurology, PD Hinduja National Hospital and Medical Research Centre, Veer Savarkar Marg, Mahim Mumbai, Maharashtra 400016 India

**Keywords:** Supportive care, Palliative care, Older adults, SPICT-LIS, LASI, Need estimation

## Abstract

**Background:**

One in five people will be older than 60 by the year 2050 in India. This demographic transition demands integration of geriatric and palliative care. The national level burden of palliative and supportive care needs of the older population is largely unknown in India. This study estimates the burden of palliative care needs among the older population in India from a nationally representative survey - Longitudinal Ageing Study of India (LASI).

**Methods:**

The general indicators of poor or deteriorating health from the Supportive and Palliative Care Indicator Tool for Low Income Setting were used to identify older adults with palliative care needs. These indicators were compared with the LASI data and matched with the appropriate variables. Descriptive statistical analysis, chi-square tests and multivariate logistic regression were done to estimate palliative care needs and its association with other characteristics.

**Results:**

12.2% of Indian older adults have supportive and palliative care needs. Among Indian states, highest for West Bengal (17%), Madhya Pradesh (16.9%), and Bihar (16.3%) while lowest in Arunachal Pradesh (2.2%), Nagaland (2.4%), and Mizoram (3%). High needs were found among those aged 70 years and above (AOR-1.86), females (AOR-1.33), Muslim religion (AOR-1.24), rural residents (AOR-1.72), those who experienced ill-treatment (OR-1.75), with cancer (AOR-2.84), respiratory disease (AOR-3.14), and stroke (AOR-2.58). Lower needs were observed with higher education (AOR-0.43) and health insurance (AOR-0.83).

**Conclusion:**

This is the first study in India that estimates the need for supportive and palliative care using a nationally representative sample. One among eight older adults in India has supportive and palliative care needs. The needs are higher among female older adults, rural residents, older adults with chronic diseases, and in poorer States. Screening and early integration of palliative care with routine healthcare care is essential to meet these needs.

## Background

The global transition in ageing demands the integration of geriatric care and palliative care [1]. Globally, the proportion of older adults aged 65 years and above is expected to rise to 16% in 2050 from 6% in 1990 [2]. Similarly, in India, the size of the older population aged 60 years and above is expected to increase to 20.8% by 2050, when one in five Indians will be older than 60 years of age [3]. In addition, the Lancet Commission reports that older adults will experience the most significant rise in serious health-related suffering (SHS) between 2016 and 2026. Given the significant increase in the older adult population and SHS related to ageing, palliative care should be an integral aspect of geriatric care [4].

Chronic diseases are the leading cause of disabilities and premature deaths among older adults [5]. More often these problems are found to be occurring together in older adults, presenting as complex health problems and multiple disabilities [6, 7]. As a consequence, the older adults suffer exacerbation of ongoing ailments, multiple hospitalizations, polypharmacy, decline in the Activities of Daily Living (ADL), decline in functional capacity, depression and other psychiatric morbidities, and poor quality of life (QOL) [6–12]. In the context of such complex needs, the World Health Organization (WHO) recommends integrating palliative care into the chronic disease management of older adults [4, 13].

Palliative care in geriatric practice aims to alleviate suffering, and improve quality of life through impeccable assessment, symptom control, goals of care discussions, shared decision-making, care coordination, and transition into community care settings wherever possible [1]. In India, access to palliative care is abysmally low [14]. One of the barriers to the integration of palliative care is that palliative care needs are not routinely screened and identified even in the presence of chronic life-limiting conditions [15]. In the context of poor screening and identification, the burden of palliative and supportive care needs is largely unknown in India. Though there are a few regional estimations of the burden of palliative care needs among the older population [16–18], a national-level estimation of the same is not available for India. This paper aims to estimate the burden of supportive and palliative care needs among the older population of India from the nationally representative LASI (Longitudinal Ageing Study of India).

## Methodology

The study used the Supportive and Palliative Care Indicator Tool-Low Income Setting (SPICT-LIS) to identify older adults with palliative care needs. The tool was developed at the University of Edinburgh by the Primary palliative care research group [19]. The tool provides both general indicators of poor or deteriorating health and disease-specific indicators to identify palliative care needs. This study used the general indicators of poor or deteriorating health from SPICT-LIS to identify older adults aging 60 years and above with supportive and palliative care needs. Supportive care is defined as “the multi-disciplinary holistic care of patients with malignant and non-malignant chronic diseases and serious illness, and those that matter to them, to ensure the best possible quality of life [20]. Palliative care is “an approach that improves the quality of life of patients (adults and children) and their families who are facing the problems associated with life-threatening illness, through the prevention and relief of suffering by means of early identification and correct assessment and treatment of pain and other problems, whether physical, psychosocial or spiritual” [13]. The components of supportive care and palliative care includes, symptom control, physical, psychosocial and spiritual support, patient and family empowerment, optimising comfort, improving function and reducing the effects of debility [13, 21]. Due to these overlapping principles, components, and goals of care, the SPICT instrument captures supportive and palliative care needs as a whole.

LASI is a nationally representative population survey of older adults aged 45 years and above. The survey exercise is intended to be carried out every two years for 25 years. The first wave of the survey was cross-sectional by design and was conducted between 2017 and 19. This included 73,396 older adults aged 45 years and above selected by a multistage stratified area probability cluster sampling design from all the states and union territories of India. The survey was a collaborative effort by the International Institute for Population Sciences (IIPS), Mumbai; National Programme for Health Care of Elderly (NPHCE); Ministry of Health and Family Welfare, New Delhi; Harvard T. H. Chan School of Public Health (HSPH), Massachusetts; and the University of Southern California (USC). The survey instrument included individual schedule, household schedule and community schedule. The primary aim of the survey is to investigate the consequence of aging in the country in the light of health, social, and economic determinants. The current study used the data from individual schedule and household schedule. The data covered in these schedules include socio-demographic and socio-economic profile, work and retirement profile, disease profile, functional health, psychosocial and cognitive assessment, social activity profile, bio-marker profile and direct health examination. A detailed information on the LASI survey is available from Longitudinal Ageing Study in India Wave-1 India Report [22].

**Fig. 1:**
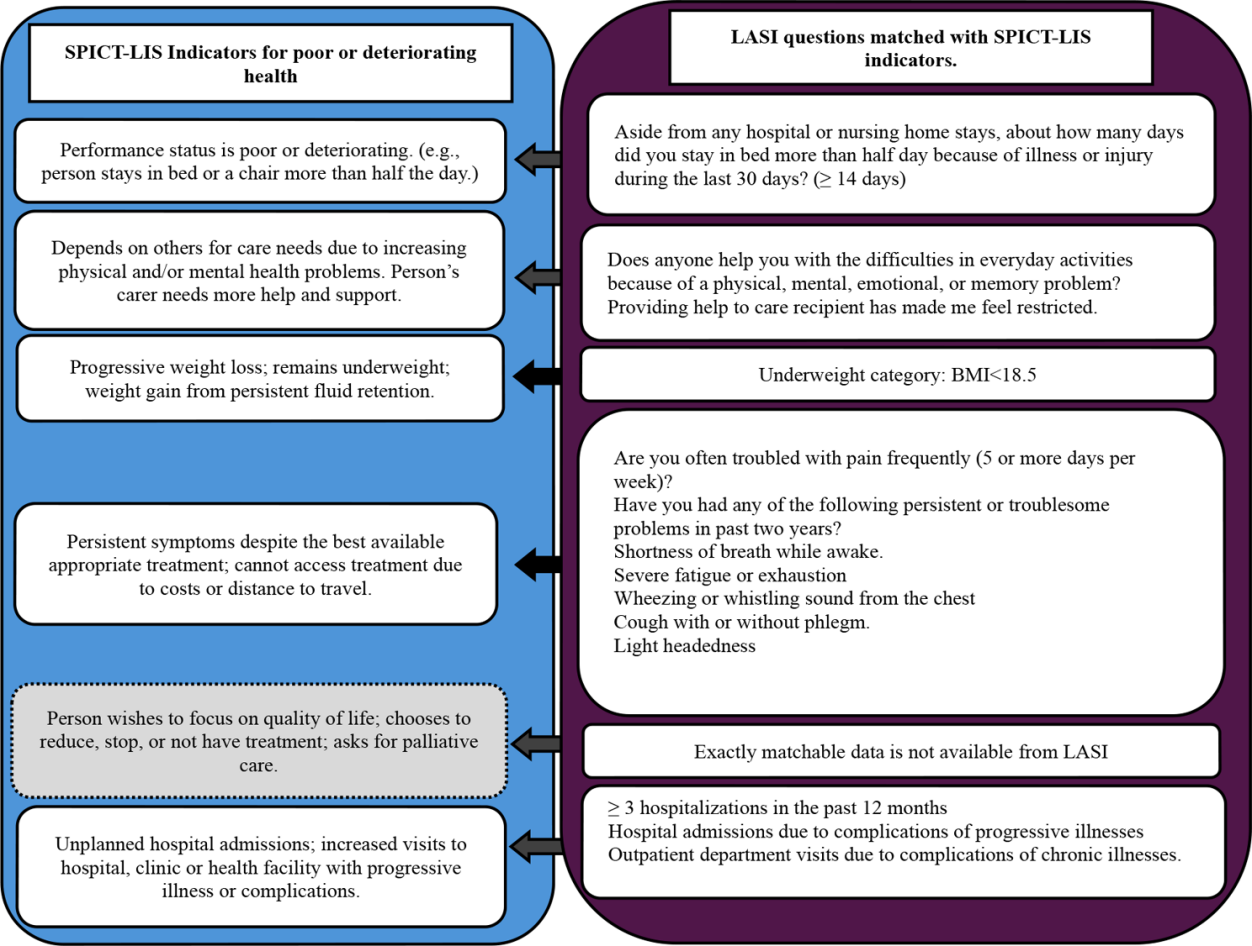
Framework for identifying elderly with supportive and palliative care needs using SPICT-LIS general indicators of poor and deteriorating health with LASI. *Source*: Authors’ mapping of SPICT-LIS criteria on Longitudinal Ageing Study in India questionnaire

The framework for matching SPICT-LIS indicators with LASI data is provided in Fig. 1. The first SPICT-LIS indicator of identifying poor performance status was matched with the data of older adults who stayed in bed for half a day or more for two weeks or more [23]. The second indicator representing the need for support and help was matched with the older adults who required support for their Activities of Daily Living (ADL) and Instrumental Activities of Daily Living (IADL). The third indicator *“Progressive weight loss; remains underweight; weight gain from persistent fluid retention”* was matched with those who belonged to the underweight category of BMI assessment. Other aspects of this indicator could not be matched in LASI data. The fourth indicator of persistent symptoms was matched with older adults who reported three or more persistent symptoms. The fifth indicator of a person’s preference for palliative care could not be mapped on LASI data. The sixth indicator of unplanned hospital admissions and visits was matched in the older adults who had multiple hospital admissions and hospital visits due to complications of life-limiting conditions.

**Fig. 2:**
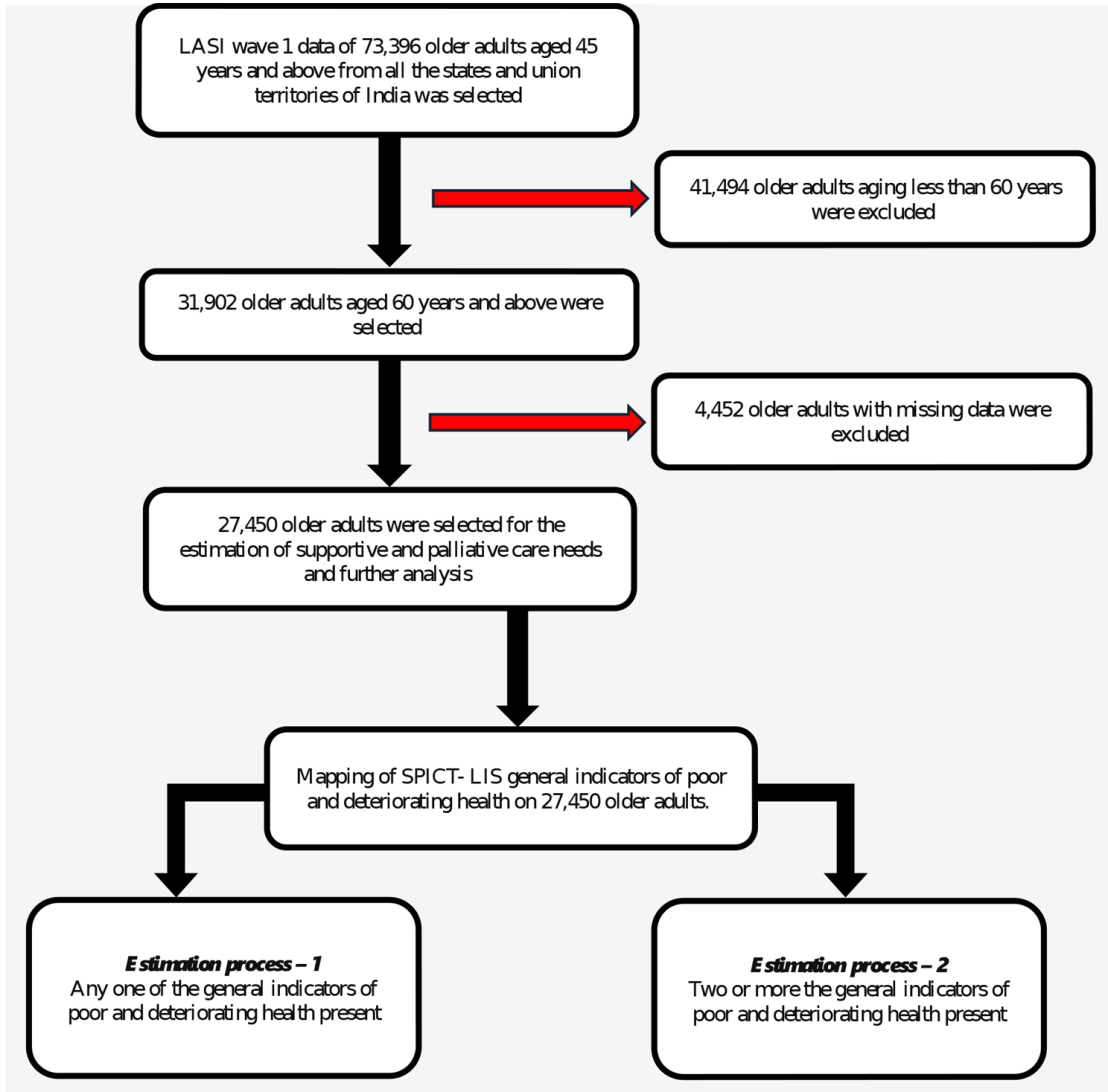
Flow diagram representing sample selection. *Source*: Authors’ description of study flow

The primary outcome variable, i.e. the need for supportive and palliative care (yes/no) was computed after mapping the SPICT-LIS general indicators of poor and deteriorating health on LASI data. The identification of those with supportive and palliative care needs was done in two ways. In the first method, older adults with any one of the general indicators of poor and deteriorating health were identified as having palliative care needs. This provides a very sensitive estimate for supportive and palliative care needs among older adults in India. In the second method, older adults were identified to have supportive and palliative care needs when they had two or more general indicators of poor and deteriorating health. Most published literature on SPICT used the second method to identify palliative care needs [24–26]. Further, the exposure variables were identified and classified by biological, social, morbidity, and risk factors profiles of older adults **(**Tables 1 and 2**)**. The respondent information with any missing data was removed before the analysis. Detailed information about the sample data selection along with the estimation process is depicted in Fig. 2.

### Statistical analysis

The study used descriptive statistics to estimate the support and palliative care needs among older adults in India. The sampling weights were applied in the estimation process as the LASI employed a multistage stratified area probability cluster sampling design, hence all the percentages provided are weighted percentages. The chi-square test was used to confirm the disparities in the palliative care needs among different sub-groups. The Multivariate logistic regression (Odds Ratio) was used for inferential statistical analysis to identify the independent association between supportive and palliative care needs and background characteristics. All the statistical analyses were performed using the statistical software package STATA - BE—Basic Edition 17, developed by Stata Corp and located in Texas 77,845, USA (StataCorp, 2021). Besides, Data Wrapper, an online GIS software was used to visualize the state-wise need for supportive and palliative care in India.

## Results

The background characteristics of older adults with supportive and palliative Care needs in India are depicted in Table 1. As shown in the table, out of 27,450 older adults aged 60 years and above considered for the analysis, 65.3% were aged between 60 and 70 years, 52.3% were females and 79.6% of them had education in primary education or below. About 43.4% of these older adults belonged to poorer or poorest quintiles while economically categorizing them into quintiles as per their household monthly per capita expenditure. Most of the older adults belonged to the Hindu religion (82.4%) and lived in rural areas (72%). Only 18.9% of the older adults had health insurance. Besides, the most common morbidity among them was hypertension (31.8%) followed by bone disease (arthritis, osteoporosis) 18.5% and diabetes (13.9%).

### Supportive and palliative care needs of older adults in India

**Fig. 3:**
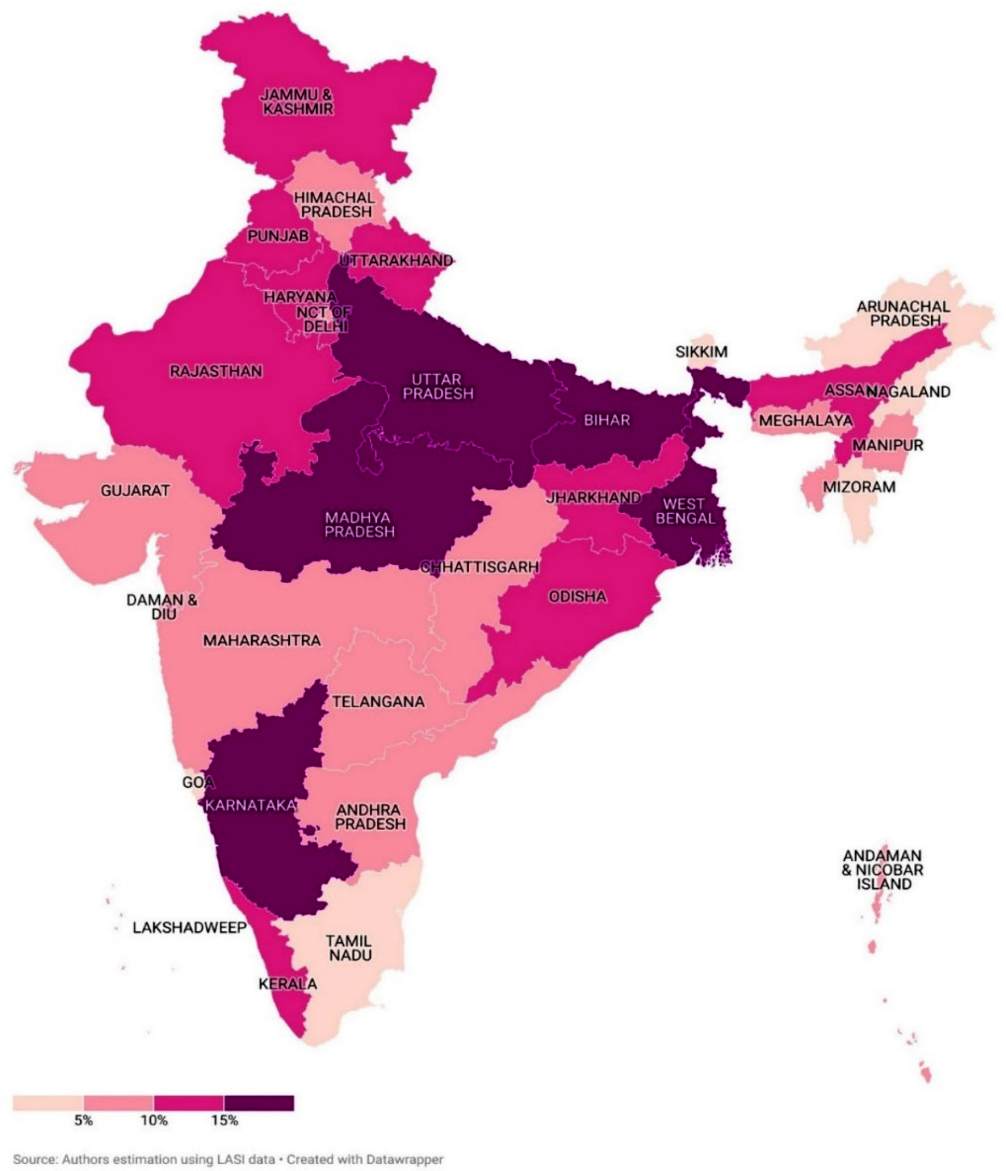
State-wise estimation of supportive and palliative care needs among older adults in India

The estimation of supportive and palliative care needs showed that 42.7% of the older adults in India had at least one SPICT-LIS general indicator for supportive and palliative care needs and 12.2% of them had two or more SPICT-LIS general indicators for supportive and palliative care needs. The mapping of supportive and palliative care needs with two or more SPICT-LIS general indicators across the Indian states is shown in Fig. 3. Among the Indian states, West Bengal had the highest supportive and palliative care needs (17%), followed by Madhya Pradesh (16.9%), Bihar (16.3%) and Uttar Pradesh (15.6%). A lower percent was observed among North-Eastern states, namely Arunachal Pradesh (2.2%), Nagaland (2.4%), Mizoram (3%), and Sikkim (3.1%).

The univariate analysis of supportive and palliative care needs with two or more SPICT-LIS general indicators showed that the older adults aged 70 years and above had higher palliative care needs (18.4%) than those aged 70 years and below (9.2%) **(**Table 1**)**. Similarly, higher palliative care needs were observed among females (13.2%) than males (11.2%). Among the education categories, higher palliative care needs were observed among those with primary education and below (13.7%) than those with higher secondary education and above (5.7%). There was no statistically significant difference in the palliative care needs between the economic categories. In the analysis of religions of older adults, a significantly higher palliative care need was observed among Muslims (15.2%) when compared with other religious categories. Higher supportive and palliative care needs were observed among the older adults who experienced ill-treatment (20.2%) and discrimination (13.9%) when compared to their counterparts.

**Fig. 4:**
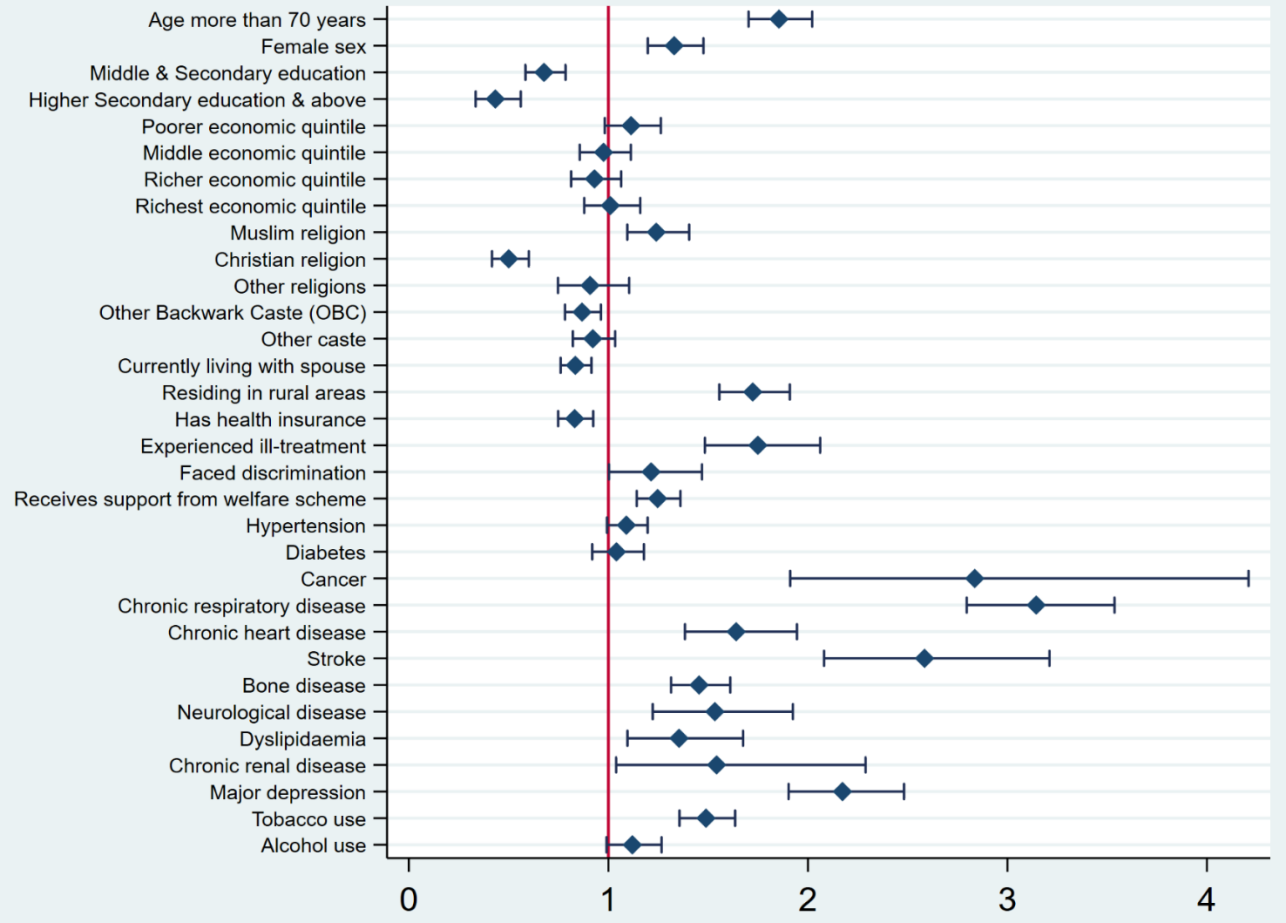
Forest plot of Multivariate Logistic Regression for Supportive and Palliative Care Needs among older adults in India. *Note*: The x-axis represents the adjusted odds ratio (AOR) for Supportive and palliative care needs with two or more SPICT-LIS general indicators. Each plot represents AOR with 95% Confidence Interval (CI). Plots of variables that do not overlap the null value 1 are statistically significant. Reference categories are provided in Table 2. *Source*: Authors’ estimation using Longitudinal Ageing Study in India (LASI) data

The association between the need for supportive and palliative care with the background characteristics of older adults by the multivariate logistic regression analysis is depicted in Fig. 4. A statistically significant higher palliative care need with two or more SPICT-LIS indicators was observed among older adults aged more than 70 years [AOR = 1.86], females [AOR = 1.33], and those living in rural areas [AOR = 1.72]. The analysis also showed an association between the experience of ill treatment [AOR = 1.75], utilization of welfare schemes[AOR = 1.25], and palliative care needs. Older adults who belonged to the Muslim religion [AOR = 1.24] had higher palliative care needs when compared with those from the Hindu religion in reference category and older adults from the Christian religion [AOR = 0.50] had lower palliative care needs when compared with those in reference category. Among the morbidity profile, higher palliative care needs were observed among older adults with chronic life-limiting conditions when compared to those who did not have these diseases respectively **(**Table 2**)**. The needs were more pronounce for those with chronic respiratory diseases [AOR = 3.14], cancers [AOR = 2.84] and stroke [AOR = 2.58]. On the other hand, the need for support and palliative care was found lower among those with Middle and secondary education [AOR = 0.68], and Higher secondary education and above [AOR = 0.43]) than those with primary education or below. The older adults covered by the health insurance scheme [AOR = 0.83] and those currently living with their spouses [AOR = 0.83] also had lower supportive and palliative care needs when compared with their counterparts.

**Table 1:** Background characteristics of older adults with supportive and Palliative Care needs in India

Description	Category	Sample frequency	Has at least one SPICT-LIS general indicator			Has two or more SPICT-LIS general indicator		
			Yes	No	*p*-value	Yes	No	*p*-value
		*n*(%)	*n*(%)	*n*(%)		*n*(%)	*n*(%)	
**India**		27,450	11,715 (42.7)	15,735 (57.3)		2803 (12.2)	24,647 (87.8)	
***Biological***								
Age	<=70	18,794 (67.3)	7302 (43.4)	11,492 (56.7)		1496 (9.2)	17,298 (90.8)	
	> 70	8656 (32.7)	4413 (55.0)	4243 (45)	0.00	1307 (18.4)	7349 (81.6)	0.00
Sex	Male	13,240 (47.7)	5266 (45.3)	7974 (54.7)		1201 (11.2)	12,039 (88.8)	
	Female	14,210 (52.3)	6449 (48.9)	7761 (51.1)	0.00	1602 (13.2)	12,608 (86.8)	0.00
***Social***								
Education	Primary & below	21,398 (79.6)	9998 (51.2)	11,400 (48.8)		2485 (13.7)	18,913 (86.3)	
	Middle & Secondary	4021 (13.4)	1257 (33.5)	2764 (66.5)		247 (6.9)	3774 (93.1)	
	Higher Secondary & above	2031 (7.0)	460 (27.7)	1571 (72.3)	0.00	71 (5.7)	1960 (94.3)	0.00
Monthly per capita Expenditure (MPCE)	Poorest	5605 (21.7)	2631 (51.7)	2974 (48.3)		574 (12.0)	5031 (88.0)	
	Poorer	5690 (21.7)	2589 (50.9)	3101 (49.2)		638 (14.0)	5052 (86.0)	
	Middle	5644 (21.1)	2353 (46.6)	3291 (53.4)		557 (12.4)	5087 (87.6)	
	Richer	5391 (18.9)	2166 (43.7)	3225 (56.4)		523 (11.7)	4868 (88.3)	
	Richest	5120 (16.6)	1976 (41.1)	3144 (58.9)	0.00	511 (10.6)	4609 (89.4)	0.07
Religion	Hindu	19,982 (82.4)	8840 (47.1)	11,142 (52.9)		2114 (11.9)	17,868 (88.1)	
	Muslim	3219 (11.0)	1422 (48.1)	1797 (51.9)		405 (15.2)	2814 (84.8)	
	Christian	2800 (2.8)	862 (43.7)	1938 (56.3)		149 (8.7)	2651 (91.3)	
	Other	1449 (3.7)	591 (47.5)	858 (52.5)	0.00	135 (12.2)	1314 (87.8)	0.00
Caste	SC/ST	9088 (27.1)	4065 (54.2)	5023 (45.8)		949 (13.5)	8139 (86.6)	
	OBC	10,487 (45.3)	4496 (45.1)	5991 (54.9)		1088 (11.6)	9399 (88.4)	
	Other	7875 (27.7)	3154 (43.6)	4721 (56.4)	0.00	766 (12.0)	7109 (88.0)	0.24
Living status	Alone/Others	10,044 (38.0)	4864 (52.0)	5180 (48)		1305 (15.0)	8739 (85.0)	
	With spouse	17,406 (62.0)	6851 (44.2)	10,555 (55.8)	0.00	1498 (10.5)	15,908 (89.5)	0.00
Residence	Urban	9115 (28.0)	2953 (35.0)	6162 (65)		627 (8.4)	8488 (91.6)	
	Rural	18,335 (72.0)	8762 (51.9)	9573 (48.1)	0.00	2176 (13.7)	16,159 (86.3)	0.00
Covered by health insurance	No	21,603 (81.1)	9234 (47.7)	12,369 (52.3)		2267 (12.6)	19,336 (87.4)	
	Yes	5847 (18.9)	2481 (44.9)	3366 (55.1)	0.66	536 (10.5)	5311 (89.5)	0.01
Experienced ill-treatment	No	26,316 (94.7)	11,032 (46.3)	15,284 (53.7)		2564 (11.8)	23,752 (88.2)	
	Yes	1134 (5.3)	683 (63.4)	451 (36.6)	0.00	239 (20.2)	895 (79.8)	0.00
Faced discrimination	No	26,383 (95.5)	11,213 (47.1)	15,170 (52.9)		2652 (12.1)	23,731 (87.9)	
	Yes	1067 (4.5)	502 (47.6)	565 (52.4)	0.01	151 (13.9)	916 (86.1)	0.00
Covered by welfare scheme	Not available	18,361 (70.0)	7207 (44.1)	11,154 (55.9)		1572 (10.7)	16,789 (89.3)	
	At least one available	9089 (30.0)	4508 (54.4)	4581 (45.6)	0.00	1231 (15.8)	7858 (84.2)	0.00
***Morbidity profile***								
Hypertension	No	18,064 (68.3)	7862 (48.5)	10,202 (51.6)		1737 (11.8)	16,327 (88.3)	
	Yes	9386 (31.8)	3853 (44.4)	5533 (55.6)	0.00	1066 (13.2)	8320 (86.8)	0.00
Diabetes	No	23,240 (86.1)	10,149 (48.7)	13,091 (51.3)		2397 (12.6)	20,843 (87.4)	
	Yes	4210 (13.9)	1566 (37.6)	2644 (62.4)	0.00	406 (9.6)	3804 (90.5)	0.18
Cancer	No	27,260 (99.3)	11,625 (47.1)	15,635 (52.9)		2761 (12.1)	24,499 (87.9)	
	Yes	190 (0.7)	90 (53.6)	100 (46.5)	0.19	42 (33.5)	148 (66.5)	0.00
Lung disease	No	25,432 (91.7)	10,466 (45.8)	14,966 (54.2)		2277 (10.9)	23,155 (89.2)	
	Yes	2018 (8.3)	1249 (62.5)	769 (37.5)	0.00	526 (27.2)	1492 (72.8)	0.00
Heart Disease	No	26,072 (94.7)	11,098 (47.3)	14,974 (52.7)		2583 (12.0)	23,489 (88.0)	
	Yes	1378 (5.3)	617 (45.0)	761 (55)	0.11	220 (15.2)	1158 (84.8)	0.00
Stroke	No	26,846 (97.7)	11,347 (46.7)	15,499 (53.3)		2666 (11.9)	24,180 (88.1)	
	Yes	604 (2.3)	368 (64.9)	236 (35.1)	0.00	137 (23.7)	467 (76.4)	0.00
Bone disease	No	22,786 (81.6)	9449 (46.0)	13,337 (54.1)		2113 (11.1)	20,673 (88.9)	
	Yes	4664 (18.5)	2266 (52.5)	2398 (47.5)	0.00	690 (17.0)	3974 (83.0)	0.00
Neurological disease	No	26,837 (97.6)	11,371 (46.9)	15,466 (53.1)		2678 (11.9)	24,159 (88.1)	
	Yes	613 (2.4)	344 (59.2)	269 (40.8)	0.00	125 (24.9)	488 (75.1)	0.00
Dyslipidaemia	No	26,418 (97.6)	11,296 (47.2)	15,122 (52.8)		2666 (12.0)	23,752 (88.0)	
	Yes	1032 (2.4)	419 (47.0)	613 (53)	0.16	137 (19.2)	895 (80.8)	0.00
Chronic kidney disease	No	27,221 (99.2)	11,600 (47.1)	15,621 (52.9)		2769 (12.2)	24,452 (87.8)	
	Yes	229 (0.8)	115 (56.8)	114 (43.2)	0.02	34 (16.1)	195 (83.9)	0.02
Major depression	No	25,706 (92.0)	10,669 (45.8)	15,037 (54.2)		2407 (11.2)	23,299 (88.8)	
	Yes	1744 (8.0)	1046 (62.8)	698 (37.2)	0.00	396 (23.5)	1348 (76.5)	0.00
***Risk factors profile***								
Tobacco use	No	16,713 (59.2)	6394 (42.6)	10,319 (57.4)		1443 (10.6)	15,270 (89.4)	
	Yes	10,737 (40.8)	5321 (53.7)	5416 (46.3)	0.00	1360 (14.5)	9377 (85.5)	0.00
Alcohol use	No	22,679 (85.0)	9548 (46.6)	13,131 (53.4)		2291 (12.2)	20,388 (87.8)	
	Yes	4771 (15.0)	2167 (50.3)	2604 (49.7)	0.00	512 (12.0)	4259 (88.00)	0.19

Source: Authors’ estimation using LASI Data

**Table 2:** Regression analysis for supportive and Palliative Care needs among older adults in India

Description	Category	Regression analysis for at least one SPICT-LIS general indicator	Regression analysis for two or more SPICT-LIS general indicator
		Adjusted OR [CI]	Adjusted OR [CI]
**Biological**			
Age	<=70	1^®^	1^®^
	> 70	1.57 [1.48–1.66]***	1.86 [1.7–2.02]***
Sex	Male	1^®^	1^®^
	Female	1.34 [1.26–1.43]***	1.33 [1.20–1.48]***
***Social***			
Education	Primary & below	1^®^	1^®^
	Middle & Secondary	0.71 [0.65–0.77]***	0.68 [0.58–0.79]***
	Higher Secondary & above	0.55 [0.49–0.62]***	0.43 [0.33–0.56]***
Monthly per capita Expenditure (MPCE)	Poorest	1^®^	1^®^
	Poorer	0.96 [0.89–1.04]	1.11 [0.98–1.26]
	Middle	0.84 [0.78–0.91]***	0.98 [0.86–1.11]
	Richer	0.79 [0.73–0.86]***	0.93 [0.81–1.06]
	Richest	0.79 [0.73–0.86]***	1.01 [0.88–1.16]
Religion	Hindu	1^®^	1^®^
	Muslim	1.03 [0.95–1.11]	1.24 [1.09–1.40]**
	Christian	0.54 [0.5–0.6]***	0.50 [0.42–0.60]***
	Other	0.93 [0.83–1.05]	0.91 [0.75–1.10]
Caste	SC/ST	1^®^	1^®^
	OBC	0.88 [0.82–0.94]***	0.87 [0.78–0.96]**
	Other	0.96 [0.89–1.03]	0.92 [0.82–1.03]
Living status	Alone/Others	1^®^	1^®^
	With spouse	0.85 [0.81–0.91]***	0.83 [0.76–0.92]***
Residence	Urban	1^®^	1^®^
	Rural	1.71 [1.61–1.81]***	1.72 [1.56–1.91]***
Covered by health insurance	No	1^®^	1^®^
	Yes	0.95 [0.89–1.01]	0.83 [0.75–0.92]**
Experienced ill-treatment	No	1^®^	1^®^
	Yes	1.58 [1.39–1.80]***	1.75 [1.48–2.06]***
Faced discrimination	No	1^®^	1^®^
	Yes	1.04 [0.92–1.19]	1.21 [1.01–1.47]*
Covered by welfare scheme	Not available	1^®^	1^®^
	At least one available	1.14 [1.08–1.21]***	1.25 [1.14–1.36]***
***Morbidity profile***			
Hypertension	No	1^®^	1^®^
	Yes	0.93 [0.87–0.98]**	1.09 [0.99–1.20]
Diabetes	No	1^®^	1^®^
	Yes	0.99 [0.92–1.08]	1.04 [0.92–1.18]
Cancer	No	1^®^	1^®^
	Yes	1.37 [1.01–1.87]*	2.84 [1.91–4.21]***
Chronic respiratory disease	No	1^®^	1^®^
	Yes	2.16 [1.96–2.38]***	3.14 [2.80–3.54]***
Heart Disease	No	1^®^	1^®^
	Yes	1.23 [1.09–1.38]**	1.64 [1.38–1.94]***
Stroke	No	1^®^	1^®^
	Yes	2.37 [1.97–2.84]***	2.58 [2.08–3.21]***
Bone disease	No	1^®^	1^®^
	Yes	1.27 [1.19–1.36]***	1.45 [1.31–1.61]***
Neurological disease	No	1^®^	1^®^
	Yes	1.47 [1.24–1.76]***	1.53 [1.22–1.92]***
Dyslipidaemia	No	1^®^	1^®^
	Yes	1.18 [1.03–1.37]*	1.35 [1.10–1.67]**
Chronic kidney disease	No	1^®^	1^®^
	Yes	1.57 [1.19–2.09]**	1.54 [1.04–2.29]*
Major depression	No	1^®^	1^®^
	Yes	1.72 [1.55–1.91]***	2.17 [1.90–2.48]***
***Risk factors profile***			
Tobacco use	No	1^®^	1^®^
	Yes	1.53 [1.45–1.63]***	1.49 [1.36–1.63]***
Alcohol use	No	1^®^	1^®^
	Yes	1.11 [1.03–1.19]**	1.12 [0.99–1.27]

Source: Authors’ estimation using LASI Data

Note: * *p* < 0.05, **p < 0.01, ***p < 0.001, OR = Odds Ratio, CI = Confidence Interval

## Discussion

The estimation of supportive and palliative care needs with any one of the SPICT-LIS general indicators of poor and deteriorating health as the identification criteria showed that 42.7% of the older adults have palliative care needs. This may be a very sensitive estimate as the threshold for supportive and palliative care needs was set at just one indicator. On reviewing the literature, only one study [27] using just one indicator to identify individuals with palliative care needs was found. The second method using two or more general indicators of poor and deteriorating health, the estimation showed that 12.2% of the Indian older adults have palliative care needs. This is a balanced estimate and most studies using SPICT used two or more general indicators to identify individuals with supportive and palliative care needs [24–26]. This estimate is similar to another study done in India that used SPICT-ALL to identify individuals with palliative care needs using a locally representative sample [24].

The current estimate shows that one in eight older adults in India have supportive and palliative care needs. As the population of older adults is projected to increase to 347 million by 2050 in India [3], this burden is likely to increase by severalfold. The healthcare providers attending to older adults at various chronic disease clinics, and geriatric clinics at all levels of the health system should screen for supportive and palliative care needs and integrate palliative care into patient care early in the course of their treatment [28–31]. To address this growing need, the Government of India launched the National Program for Palliative Care (NPPC) in the year 2012 which offers funding support for states to develop and implement palliative care programs [32].

Among the Indian states, a higher proportion of palliative care needs among older adults was observed in West Bengal, followed by Madhya Pradesh and Bihar. The LASI report shows that the frailty indicators such as restrictions in activities of daily living and being underweight were observed in higher numbers in these states [22]. In addition, West Bengal has the highest prevalence of stroke and neurological illnesses among the Indian states [33]. It is important to note that, among the 28 states and 8 union territories, a state palliative care policy is available only in states such as Kerala, Tamil Nadu, Karnataka, and Maharashtra [34].

This nationally representative sample shows that 72% of the older adults in India reside in rural areas, and 13.7% of them require palliative care, whereas only 8.4% of the older adults in urban areas have such needs. Other studies also show that the palliative care needs among rural areas in India are high [35–37]. Palliative care provisions in India are isolated [13] and in rural areas, they are severely limited [38]. Only the state of Kerala has successfully integrated palliative care services into rural panchayats (local government administration bodies) [39] showing integrating palliative care into primary health care could be a significant step towards bridging this gap [40].

Religion emerged as one of the variables that was independently associated with palliative care needs. Specifically, older adults who belonged to the Muslim community had higher palliative care needs when compared with the Hindu religion. The LASI report revealed a significantly higher proportion of older adults in this community had a higher prevalence of heart disease and lung disease and impairments in ADL and IADL requiring support from caregivers [22].

Supportive and palliative care needs were high for older adults with chronic life-limiting conditions. Among them, the needs were significantly higher for chronic respiratory diseases, followed by cancers and stroke. Palliative care needs in chronic respiratory conditions tend to become more pronounced with advancing age [13] it usually coexists with frailty [41] and higher symptom burden impairing QOL [42]. Similarly, older adults with stroke tend to have residual paralysis, cognitive impairment, and other deficits with high dependence [43, 44]. In India, stroke and its subsequent impairments emerged as significant contributors to palliative care needs [16, 24, 35].

The supportive and palliative care needs are higher among older adults who experienced ill-treatment and among those with major depression. Older adults with palliative care needs have a high level of dependency. In most cases, family members are the immediate care providers, and they are also the perpetrators of ill-treatment [22, 45]. Caregiver stress, burden, and anxiety are significant predictors of abuse [46]. Palliative care seeks not only to improve the QOL of the patients but also offers a strong support system for the family caregivers [13] and can play a pivotal role in addressing ill-treatment experienced by older adults. Furthermore, studies show that palliative care needs and major depression tend to coexist especially among older adults [47–49]. These findings underpin the importance of screening older adults for depression, especially the ones with palliative care needs [50, 51].

The current analysis showed significantly higher palliative care needs among older adults with primary level education or less when compared to those with higher education. A similar finding was reported in another community-based study [52]. In exploring the reasons, the LASI report revealed that the treatment rates for chronic life-limiting illnesses were significantly lower among those with lower levels of education [22]. Poor treatment rates in life-limiting illnesses increase the risk of complications [53] and can explain higher palliative care needs among older adults with lower education levels.

### Strengths and limitations

This is the first study that provides a national-level estimate for supportive and palliative care needs among older adults in India using a nationally representative sample survey with good external validity. The SPICT-LIS is a validated instrument in identifying individuals with supportive and palliative care needs, this adds merit to the current estimate. Nevertheless, the LASI data does not have information on the fifth SPICT-LIS indicator “Person wishes to focus on quality of life; chooses to reduce, stop or not have treatment; asks for palliative care”, and the information regarding “progressive weight loss” and “persistent fluid retention” in the third indicator. The absence of this information may be a limitation of the current study. The limitations of the LASI survey such as respondent fatigue as a consequence of lengthy survey and local dialect challenges applies to the current paper.

## Conclusion

One among eight older adults in India has supportive and palliative care needs. Palliative care needs among older adults are higher among females, those who live in rural areas, and those with lower education levels. Higher palliative care need was observed among older adults with chronic life-limiting conditions, yet the need was more pronounced among older adults with chronic respiratory conditions, stroke, and cancers. Older adults with major depression and who experienced ill treatment have higher palliative care needs. Screening for palliative care needs in geriatric practice and chronic disease clinics and early integration with palliative care services can help address these unmet needs. As the proportion of older adults in India is estimated to grow several folds along with the increase in serious health-related suffering among them, the integration of geriatric services, and palliative care services into primary health care is of paramount importance.

## Data Availability

The dataset used in the current study is available for download on request from the website of International Institute of Population Sciences, Mumbai. The data can be requested using the following link. https://www.iipsindia.ac.in/content/LASI-data.

## Abbreviations

SHS: Serious health–related suffering
ADL: Activities of Daily Living
WHO: World Health Organization
QOL: Quality of Life
LASI: Longitudinal Ageing Study of India
SPICT: LIS–Supportive and Palliative Care Indicator Tool–Low Income Setting
IADL: Instrumental Activities of Daily Living
IIPS: International Institute for Population Sciences
NPHCE: National Programme for Health Care of Elderly
HSPH: Harvard T. H. Chan School of Public Health
USC: University of Southern California
BMI: Body Mass Index
CIDI: SF–Short Form Composite International Diagnostic Interview
GIS: Geographic Information System
OBC: Other Backward Caste
AOR: Adjusted Odds Ratio
MPCE: Monthly per capita Expenditure
SC/ST: Scheduled Caste/Scheduled Tribe
NPPC: National Program for Palliative Care\
